# Preparing an Intervention to Increase Engagement with the 988 Suicide and Crisis Lifeline Resource among Community Mental Health Consumers with Schizophrenia Spectrum Disorders

**DOI:** 10.1007/s10597-026-01599-5

**Published:** 2026-02-27

**Authors:** Lindsay A. Bornheimer, Adrienne Lapidos, Nicholas M. Brdar, Alexandra N. Kelter, Jonathan Song, Ganesh Palaniappan, Chloe Miner, Maura Campbell, Katie Hoener, Timothy Florence, Mark A. Ilgen

**Affiliations:** 1 University of Michigan–Ann Arbor, Ann Arbor, USA; 2 Washtenaw County Community Mental Health, Ypsilanti, USA; 3 Department of Veteran Affairs Center for Clinical Management Research, VA Ann Arbor Health Care System, Ann Arbor, USA

**Keywords:** Crisis line facilitation, Suicide prevention, Crisis resource, Schizophrenia spectrum disorders, Psychosis

## Abstract

Suicide rates are high among individuals with schizophrenia spectrum disorders (SSDs) and community mental health (CMH) settings have potential to play a critical role in suicide prevention efforts. Crisis lines, such as the 988 Lifeline, are found to positively impact mental health and suicide outcomes, yet data show utilization rates are low among high-risk individuals, such as those with SSDs. This qualitative study employs community-engaged methods to modify a brief single-session Crisis Line Facilitation (CLF) intervention for individuals with SSDs in CMH to increase the likelihood of engagement with the 988 Lifeline and reduce suicide outcomes. Participants (*n* = 10) included consumers with SSD diagnosis, mental health providers, peer support specialists, and leadership in CMH. Data were collected in qualitative interviews to learn about perceptions of CLF, barriers and facilitators of CLF implementation, and suggested modifications to CLF for SSD tailoring. Interview questions were informed by the Consolidated Framework for Implementation Research and a hybrid analytic approach with inductive and deductive coding was used to identify themes and subsequent modifications. Themes pertained to the need for CLF in CMH, help-seeking and psychosis symptoms as implementation barriers, and provider preparation and CMH awareness as implementation facilitators. Modifications and study implications signal the importance of provider training, proactively addressing consumer help-seeking barriers, and embedding CLF within routine CMH workflows for sustainability. Study efforts emphasize CLF’s potential to prepare SSD consumers for suicide crises, motivate future 988 engagement, and prevent premature suicide death.

## Introduction

The rate of suicide death in the United States have increased by 35% from 1999 to 2022 (Martínez-Alés et al., 2022). Notably, suicide is the largest contributor to heightened premature mortality among individuals with schizophrenia spectrum disorders (SSDs; Sher et al., 2019), with data showing a lifetime rate of suicide death being approximately 20 times greater than that of the general population (Nordentoft et al., 2013; Too et al., 2019; Zaheer et al., 2020). Despite the well-documented risk for suicide among individuals with SSDs, there remains a gap in suicide prevention-focused interventions and a need for tailoring efforts to better meet specific psychosis symptom and SSD illness experiences (Bornheimer et al., 2020; Chong et al., 2020; Comendador et al., 2023; Sicotte et al., 2021; DeVylder et al., 2015). Given community mental health (CMH) settings are among the largest mental health providers in the United States and deliver the majority of care to individuals with SSDs (Adamou, 2005), it is critical that interventions are designed for and delivered to consumers with SSDs in CMH.

A longstanding approach to suicide prevention involves the use of emergency crisis hotlines that aim to provide emotional support, crisis intervention, and resources to help individuals in distress (Litman et al., 1965; Shneidman et al., 1956). Crisis lines, such as the 988 Suicide and Crisis Lifeline, are considered a best-practice approach within the CDC’s technical package of programs and practices for suicide prevention (Stone et al., 2017). A recent study indicates that the vast majority of individuals who engaged with the 988 Lifeline during a suicide-related crisis found the call to be helpful (98%) and stopped them from engaging in suicide behavior (88.1%; Zaheer et al., 2020), providing strong evidence of 988’s utility. Although literature shows support for crisis line use in reductions of suicidal ideation, hopelessness, psychological pain, and psychological distress (Kilbourne et al., 2013; McCarthy et al., 2009), data also indicate that only approximately 5% of people experiencing distress have used a crisis line (Purtle et al., 2023). Ultimately, the effectiveness of crisis lines is limited by low rates of use from individuals who could benefit most from the resource (Ilgen et al., 2022), such as the high suicide-risk SSD population.

Crisis Line Facilitation (CLF) is an intervention to increase use of the 988 Lifeline during periods of high suicide risk. Originally designed and delivered in the Veteran Affairs (VA) healthcare system with more recent modifications made for substance use treatment settings (Ilgen et al., 2022), CLF is a single-session intervention that involves components of Motivational Interviewing and behavioral rehearsal to encourage use of 988 during a crisis (Ilgen et al., 2023). The intervention involves four core steps: (1) review of a prior suicide crisis (i.e., ideation and attempt), (2) 988 psychoeducation, (3) discussion of barriers and facilitators of 988 use, and (4) a 988 practice call and processing of the experience. Although CLF literature indicates that participants perceive the intervention to be helpful, express greater comfort and confidence in calling 988 when in crisis, and report fewer suicide attempts after the intervention (Ilgen et al., 2022, 2023), it is unknown if CLF is appropriate and useful for individuals with SSDs receiving care in CMH settings.

Modifications to the CLF intervention are needed prior to delivery in CMH settings among consumers with SSDs given distinct psychosis symptoms, illness-related experiences, and barriers to help-seeking contribute to suicide risk differently as compared to other mental health service users. In particular, symptom-specific risk factors differ from other service users given specific psychosis symptoms (e.g., auditory hallucinations or paranoid delusions) are known to increase suicide thoughts and behavior (Bornheimer et al., 2020). Furthermore, many people with SSD diagnoses have treatment-related psychological trauma from illness-related experiences (e.g., involuntary hospitalization, seclusion) that can influence reasons for avoiding care in times of crisis (Chieze et al., 2019; Hallett et al., 2024). As a result, and to increase the reach of the 988 Lifeline resource as a suicide prevention approach, this qualitative study aims to modify the CLF intervention for a new population and setting for future delivery among consumers with SSDs in CMH. To inform CLF modifications, this study sought to collect qualitative data from consumers, providers, peers, and leadership in CMH regarding the need for CLF, barriers for CLF implementation, and facilitators for CLF implementation.

## Methods

Qualitative data were collected in a CMH location in the midwestern United States to inform CLF modifications. This specific CMH setting provides the vast majority of public mental health services in a state with a suicide rate of 14.6 deaths per 100,000 total population, which is a 47% increase from 2000 to 2022 (Michigan Department of Health and Human Services, 2023). The Consolidated Framework for Implementation Research (CFIR; Damschroder et al., 2009) was used to guide qualitative interview design and data analysis in preparation for intervention modifications. This study was approved by the Institutional Review Board at the University of Michigan.

### Participants

A total of 10 participants enrolled to engage in qualitative data collection, including 3 consumers and 7 staff (2 peer support specialists, 3 mental health providers, and 2 leadership members including program managers and directors) at the CMH setting. Eligibility for consumers included the following criteria: (1) age of 18 or older, (2) current engagement with CMH services (i.e., active consumers), (3) diagnosis of a schizophrenia spectrum disorder (SSD), and (4) endorsement of suicidal ideation or attempt within 6 months of screening based upon items of the Columbia-Suicide Severity Rating Scale (C-SSRS; Posner et al., 2011). Eligibility for staff participants (mental health providers, peers, and leadership) included the following criteria: (1) age of 18 years or older, (2) employed by the CMH setting, and (3) having direct contact with and/or providing clinical supervision or oversight to consumers with SSDs.

### Study Procedures

Recruitment occurred through informational flyers and emails that were sent to CMH providers, peers, and leadership from members of the research team. Emails shared details of the study and described steps to take if interested in contributing as a participant. Once informed consent was obtained, participants were screened using criteria described above with research staff. Eligible participants completed a brief survey of participant characteristics, subsequently learned about the existing CLF intervention using a short presentation by research staff, and ultimately attended an in-depth qualitative interview with research staff. Research staff included trained undergraduate and graduate-level study coordinators and research assistants in social work, psychology, and psychiatry fields within in the lead author’s research lab. The brief survey of participant characteristics pertained to demographics (e.g., age, gender, race, etc.), clinical experiences for consumers (e.g., diagnosis, service use, experience with 988 and crisis resources, etc.), and professional experiences for staff (e.g., field of practice, license type, 988 use among consumers, years at CMH, role, scope of work, etc.).

Qualitative in-depth interviews were conducted by research staff, recorded using videoconferencing software, and lasted for 30–60 min. Interview questions were guided by CIFR (Damschroder et al., 2009; Reardon et al., 2025) and sought to explore barriers and facilitators of the CLF intervention and its implementation in CMH for consumers with SSDs within domains of intervention characteristics (e.g., core components and adaptable elements), the outer setting (e.g., economic, political, and social context surrounding the organization), inner setting (e.g., the aforementioned contexts through which the implementation proceeds), individual characteristics (e.g., of those who play a key role in intervention and/or implementation), and implementation process (e.g., active change that contributes to implementation and may involve multilevel simultaneous sub-processes). Participant perceptions were explored using spontaneous probing for expansion of experiences and perceptions (Foley & Timonen, 2015) with probes existing within the interview guide to allow for real-time exploration for greater understanding (Corbin & Strauss, 2008). Probe examples include: *If clarification is needed*, “what, if anything, might help increase the likelihood of this 988-use intervention being delivered to consumers in community mental health?” Also, *if organizational factors were only mentioned and not consumer- or provider-level factors*, “is there anything that comes to mind about consumer experiences or mental health providers that could possibly get in the way of this intervention being delivered?”

### Data Analysis

Descriptive explorations were conducted in SPSS28 to convey stakeholder characteristics. Qualitative interview data were transcribed by trained research staff and a hybrid approach to thematic analysis (Fereday & Muir-Cochrane, 2006; Swain, 2018) was used involving both deductive and inductive coding procedures guided by CFIR domains (Damschroder et al., 2009). Two research staff were trained to code and independently engaged in content analysis of interview data in the inductive step, allowing for two *processes of discovery* and *fine tuning* any a priori sensitizing theories and concepts (Morgan, 1996). Consensus was reached by the coders and the first author after content analysis was completed regarding themes that emerged within the qualitative question categories (Boyatzis, 1998). A final matrix was generated to understand and convey findings. The study used the following qualitative strategies of rigor (Padgett, 2016; Padgett, 2017; Hamilton et al., 2018) to ensure robust and unbiased results: (1) audit trail, (2) analytic triangulation, and (3) debriefing meetings.

## Results

### Participant Characteristics

Table 1 presents characteristics of consumer and staff participants. All consumers had a diagnosis of schizoaffective disorder and 19.67 years (SD = 4.51) was the mean duration of lifetime illness. Staff had, on average, 8.71 years of experience in the mental health field (SD = 5.22) and have been working for 5.85 years at this specific CMH location (SD = 5.18).

**Table 1:** Participant characteristics

	*n*	%
Age (M, SD)	10	42.34, 7.09
Sex		
Female	7	70%
Male	3	30%
Race		
White	8	80%
Black	2	20%
Ethnicity		
Hispanic/Latino	0	0%
Not Hispanic/Latino	9	90%
Unknown	1	10%
Highest Education Level Completed		
High School Graduate	2	20%
Some College Attended	2	20%
College Graduate	1	10%
Graduate/Professional School	5	50%
Employment Status		
Unemployed	2	20%
Working Full-Time	7	70%
Retired	1	10%
Consumer Use of 988 Lifeline^a^		
Never used	2	66.7%
Used once	1	33.3%
Staff Years in Mental Health Field (M, SD)^b^	8.71	5.22
Staff Years at CMH (M, SD) ^b^	5.85	5.12
Staff Perception of 988 Helpfulness for Consumers ^b^		
Helpful	3	42.9%
Not Helpful	1	14.3%
No Experience with Clients using 988	3	42.9%

^a^ Consumers at CMH *n* = 3

^b^ Staff at CMH *n* = 7

### Qualitative Themes

Themes and descriptive codes are presented in a matrix (Table 2) organized by theme categories: CLF intervention perceptions, implementation barriers, and implementation facilitators.

**Table 2:** Qualitative themes and descriptive codes of participants

Theme Categories	Themes	Descriptive Codes
Perceptions about the CLF intervention	Intervention is needed in CMH	limited suicide prevention services at CMH, limited weekend resources, minimal local 988 awareness, need efforts beyond national recognition, intervention useful amid rising demand and shrinking resources
	Intervention has potential to be effective	intervention supports crisis management, importance of crisis preparation, potential to save lives, need to practice prior to a crisis, positive/helpful intervention, crisis line hesitation due to hospitalization fears, intervention fosters hope, strength, and suicide-prevention tools
	Positive traits of the intervention	establishes expectations for 988 use, centers prior crisis experiences, identifies past coping strategies, brief duration (1 hour), psychoeducation-based, complements safety planning, strong intervention structure, practice call reduces fear/anxiety about reaching out
	Tailoring for psychosis is needed	heterogeneity in psychosis and suicidal experiences, intervention response varies by symptoms, psychosis is an alternative reality experience, hallucinations elevate suicide risk, psychosis complicates 988 engagement, symptoms interfere with help-seeking, voices may undermine insight, 988 not designed for psychosis, amplified fears of police/hospitalization, need for psychosis-specific tailoring, CMH training for providers is largely general, difficulty with reality testing in a psychosis-related suicide crises
Implementation barriers for CLF	Help-seeking perceptions and stigma	suicide stigma, many people hide suicidal thoughts, ambivalence toward help-seeking, fear of being perceived as something wrong, lack of insight into need for help, cognitive entrapment in suicidal thinking, fear of reaching out, police-related fears affecting help-seeking, difficulty discussing suicide with providers
	Psychosis-related challenges	impaired reality testing limits help-seeking, difficulty recognizing crisis onset, psychosis symptoms obstruct help-seeking, impaired clinical insight is a barrier, need for greater provider knowledge of psychosis–suicide dynamics, limited symptom recognition for some consumers, shame and stigma around psychosis and suicide, psychosis acuity as a challenge
	Organizational and logistical concerns	reduced relevance for homeless consumers, limited phone access for 988, time constraints for intervention participation, need for leadership buy-in, additional provider training needed, CMH staff turnover, competing CMH programming, information overload from paperwork/brochures, transportation barriers, unstable consumer schedules
Implementation facilitators for CLF	Provider preparation	enhanced suicide prevention skills through training, engagement skills for psychosis and suicide, increased provider empathy, training needed for CMH providers, preparation to tailor intervention for various psychosis symptoms, provider readiness for difficult suicide conversations, rapport-building skills to mitigate paranoia-related barriers
	Increasing awareness	low consumer awareness of 988, need for friend and family knowledge of intervention and 988, limited awareness of 988 as a local resource, value of take-home materials to reinforce 988 awareness and intervention skill use
	Organizational integration and programmatic support	group delivery to expand reach, potential follow-up beyond one session, leadership buy-in, strengthened 988–CMH partnership, integration into existing appointments (e.g., intake, medication review)

### CLF Intervention Perceptions

Four specific themes emerged regarding positive perceptions, including: (1) the intervention is needed in CMH, (2) the intervention has potential to be effective, (3) the intervention has positive traits, and (4) tailoring is necessary for individuals experiencing psychosis. First, participants unanimously viewed CLF as filling a critical gap in suicide prevention services at CMH. The majority further described limited or inconsistent suicide-specific programming in the CMH location and emphasized that consumers often lack accessible crisis-support options. Awareness of 988 was noted to be low among both consumer and staff participants, reinforcing a perceived need for structured guidance for consumers to be more prepared for when and how to use the 988 resource. A staff reflected on this gap:

*I don’t feel like there’s like a whole lot of suicide prevention happening on like a larger scale, like an initiative in this area. Of course there are signs all over saying like, you know, 988, but I think it’s kind of the end there with the signs…like, do you even know what 988 is? Do you even know what these numbers mean and what happens when you call?*

Second, participants consistently described CLF as a practical and actionable approach that could help consumers manage suicidal crises more effectively. Several strengths of the intervention include the brevity of the one-hour format, opportunities to practice reaching out to 988, guidance on what to expect when using the resource, and the incorporation of consumers’ prior crisis experiences to prepare one for future use. All provider participants noted that practicing a call to 988 and identifying strategies that helped in past crises could build confidence and preparedness for consumers. A participant reflected on the helpfulness of a practice call in the intervention:

*I like the idea of practicing a call, so folks know what that looks like, what that feels like. I think that’s important. I do think there’s often a fear or anxiety of just making that call and not knowing what to expect when the person picks up on the other line.*

Third, participants unanimously conveyed that psychosis introduces unique challenges during suicide crises, making tailored content essential in CLF modifications. Many described psychosis as an experience that alters the perception of reality, negatively impacts clinical insight, and increases vulnerability to having suicide thoughts, all of which are important factors that could impede help-seeking and use of the 988 resource. Participants also stressed concerns that consumers often fear police or hospitalization and some 988 responders may not be well-equipped to engage individuals with psychosis. Staff further described that CMH’s current training is not sufficiently specialized in suicide prevention for psychosis, strengthening support for a more tailored intervention approach. Two participants reflected on the uniqueness of psychosis symptoms and fears of police involvement, respectively:

*Being psychotic… I just say it’s like being in another world. You’re not even in the same world. So it would be important to find ways to reach someone that’s not in their regular world.*

*A lot of our individuals with active psychosis struggle with reaching out*,* due to the potential of police intervention*,* and that those experiences have not been comfortable before*,* and they have been restrained in hospital settings…so those memories are strong.*

### CLF Implementation Barriers

Three themes emerged in relation to CLF implementation barriers: (1) help-seeking perceptions and stigma, (2) psychosis-related challenges, and (3) organizational and logistical concerns. First, participants unanimously described the issue of stigma surrounding suicide thoughts and subsequent impact on reduced help-seeking behavior. Shame, fear of judgment, and discomfort in discussing suicide with providers was expanded upon as barriers for consumers to reach out for help during the high-risk moment a suicide crisis. The topic of stigma as a barrier to CLF delivery also brought about similar discussion and descriptive codes to the above regarding fear of police and hospitalization. A participant reflected on the following in relation to stigma:

*It’s like there’s a lot of stigma around suicide and having suicidal thoughts. I just think people naturally try to hide something like that because they feel ashamed…but it’s not, it’s not something that you should have to feel ashamed about. You should feel open to talking about it so you can receive help and feel a little better.*

Second, participants discussed the challenge of psychosis symptoms in general, in relation to suicide risk, and engagement with 988. More specifically, participants described impairments in clinical insight, difficulty recognizing the onset of a crisis and suicide risk, and symptoms like disorganization or hallucinations impairing perceptions and reality. It was notable that all consumers expanded on their own experiences of not thinking about seeking help or mental health services when symptomatic, and staff participants echoed their own observations of suicide risk awareness and help-seeking impairments for consumers in acute phases of psychosis. A participant reflected on the psychosis-specific impact on awareness and help-seeking:

*I just think an awful lot of people that have hallucinations and stuff, sometimes they can have a really hard time believing it’s not real. And because of that it makes it really difficult for people to go and seek help when they don’t think there’s anything wrong.*

Third, participants described several CMH-specific challenges for the delivery of CLF: provider turnover, competing programmatic demands, limited time for provider training, and the difficulty of adding more to already full workflows. It is notable that all staff expressed concern about provider availability due to turnover and time that can be dedicated to learning and delivering a new intervention. Logistically, consumers spoke of concerns that homelessness or lack of phone access could limit the utility of an intervention that is centered around calling 988. This concern was described as being not only related to tools needed to reach out to 988 in a crisis but also things needed to receive the CLF intervention, such as transportation to a CMH location. A participant reflected on the following:

*A lot of people we work with are unhoused and they typically don’t have a phone. And so, and sometimes even transportation to get to CMH for services is difficult. Those are real barriers and sometimes we just have to go and try to meet with the person at places that they may frequent or a shelter that they go to…you know, really, it’s just being able to actually work out a time to see them.*

### CLF Implementation Facilitators

Three themes emerged regarding implementation facilitators participants: (1) provider preparation, (2) increasing awareness, and (3) organizational integration and programmatic support. First, both consumer and staff participants noted the impotence of provider training in preparation for CLF delivery. More specifically, staff participants discussed the need for providers to have suicide-specific skills, engage effectively with consumers experiencing psychosis, and build strong rapport to mitigate the potential of paranoia and mistrust of crisis services. Similar to what was conveyed regarding unique psychosis symptom experiences in prior themes, staff participants also emphasized the need for CLF training to prepare providers for working with fluctuating psychosis symptoms and suicide risk. Consumers expanded on the topic of provider preparations to add a desire for providers to be authentically empathetic for consumers to feel seen and heard. A participant reflected on the need for provider training:

*Providers need engagement skills so consumers feel safe and comfortable…if people don’t feel like they are being listened to without judgment then they won’t open up to a provider. A lot of those skills [engagement] need to be learned and refined over years, and that is why training is so important.*

Second, participants unanimously viewed awareness-building as central to CLF implementation success. Consumers noted they would be more likely to participate if they understood the purpose and potential benefits of CLF. Furthermore, consumers shared that since they were unfamiliar with 988 prior to this study, they would not have sought to receive CLF because it wouldn’t feel applicable. Staff similarly focused on the importance of increasing understanding of the 988 resource in real-time with building awareness about CLF as a service. Suggestions were provided by many participants about potential helpfulness of take-home materials that can be used to reinforce 988 and CLF knowledge. Although brochures were heavily mentioned, it is also important to consider the amount of physical content CMH consumers receive across all services. A participant reflected on the following issue of handout volume:

*I think without a pamphlet that would be challenging for someone to remember like, you know, what happened in the session… A lot of people…they end up getting a lot of brochures and paper work and it’s either hard to keep up with it or they just end up tossing it and so I think something that they can… stick somewhere that stands out so someone can access it easily and utilize it.*

Third, many participants touched upon the importance of organizational factors in the implementation success of an intervention. Conversations were focused on leadership support and buy-in for CLF to be valued and prioritized as a service. Related to prioritization, staff expanded on layers of commitment from leadership related to time for providers to engage with CLF training, support of providers in CLF delivery including supervision, and a process to identify consumers who will receive CLF as a service. Discussion of delivery timing and intended audience (i.e., depending on suicide risk and psychosis symptoms) led to a suggestion to embed CLF into existing CMH workflows to facilitate adoption. Specifically, a participant shared the following:

*It [the intervention] needs to be embedded in protocols, like in new provider orientation or like every six months at a staff meeting they talk about it [the intervention] and they do training. Then perhaps the delivery could be like, the actual delivery to the consumers could be a part of a yearly biopsychosocial assessment meeting or like the intake appointment that a new consumer has after referral to CMH. Kind of embedding it in a meeting or an appointment that they already have would be good…and it can just be as routine as a yearly assessment or other medication reviews or something like that.*

### CLF Modifications

Qualitative themes were reviewed amongst the investigative team and discussed in collaboration with CMH leadership to identify and agree upon changes. Modifications were made by the investigative team to the CLF training materials, the treatment protocol, and post-intervention informational take-home brochure for future testing in a pilot trial. First, a suicide risk factor checklist will be developed with specificity for psychosis experiences (e.g., “The voices get louder and more intense,” “My motivation gets really low,” or “I start worrying that people around me want to harm me”) given the emerging theme of tailoring for psychosis being warranted. This checklist will help facilitate a conversation between the consumer and provider to delve into individualized symptoms and suicide risk factors that are experienced. Second, a visual aid that shows a non-linear model of psychosis recovery will be developed given the emerging theme of psychosis symptoms as barriers. This will help facilitate a conversation between consumer and provider that identifies where vulnerability to suicide ideation may lie in the course of recovery and prepare providers to be mindful of clinical insight and subsequently tailor as needed for the delivery of CLF. For example, is post-hospitalization a key moment of vulnerability? Or is it a time of increased intensity of auditory hallucinations? The consumer will be given space to reflect on their history and identify *when* more support could be needed in a non-linear recovery process that aligns with their insight of symptoms and illness.

Third, providers will be prepared to engage in a conversation on ambivalence or reluctance about calling 988 related to past negative experiences with hospitalization or other mental health treatment and center the conversation on the consumer’s ability to make autonomous decisions. This modification connects to the emerging theme of help-seeking perceptions and stigma as implementation barriers. Providers will be trained to discuss ways to increase privacy during a call, such as using a pseudonym, and emphasize that calling 988 has the potential to increase their autonomy by averting a suicide crisis which could lead to a loss of freedom if unaddressed. Nevertheless, the decision to call 988 will be framed as belonging to the consumer alone, and barriers of privacy and autonomy will be respected. Fourth, and related to the emerging theme of help-seeking and stigma as implementation barriers, providers will be prepared to engage in discussion of how mental health stigma impacts help-seeking when experiencing suicide ideation. Fifth, a comprehensive CLF training for clinicians will be developed given codes of provider preparation within implementation barrier and facilitator themes. The training will include specific education on SSD recovery, psychosis symptom-specific suicide risk factors, stigma, and the need for respecting consumers’ concerns about loss of autonomy or past negative experiences of hospitalization and other treatment. Buy-in for the CLF intervention also has potential to be positively impacted after the establishment of a comprehensive training that is mass-delivered across this CMH site, given that stakeholder participants expressed the need for suicide prevention training and efforts among SSD consumers. Lastly, informational brochures will be developed and provided to consumers as take-home materials given the emerging theme of awareness as an implementation facilitator. These materials will be developed in collaboration with CMH stakeholders prior to future implementation and will include information to supplement the CLF intervention as a reminder of content covered and also include 988 Lifeline information.

## Discussion

Although the 988 Lifeline has been shown to be helpful and decrease distress, hopelessness, suicide ideation, and behavior (Britton et al., 2022; Gould et al., 2025; Pauwels et al., 2025), low utilization rates emphasize the need to reduce barriers for use, especially for the high suicide-risk SSD population. CLF is a promising intervention aiming to increase the utility and reach of the 988 Lifeline yet required modifications from its original development prior to delivery among consumers with SSDs in a CMH setting. Qualitative themes from participant interviews indicated a strong desire and need for CLF in CMH, with CLF implementation barriers being identified as help-seeking perceptions and stigma, psychosis-related challenges, and organizational and logistical concerns. Notably, and within the theme of stigma as a barrier to CLF implementation, a fear of coerced treatment is well-understood as a deterrent to help-seeking (Anderson et al., 2025; Swartz et al., 2003) and the anticipated loss of autonomy when engaging in 988 may weaken trust in the resource and drive hesitancy to utilize it. Facilitators of CLF implementation were identified as provider preparation, increasing awareness of CLF and the 988 resource, and organizational integration and programmatic support.

Based on qualitative findings and in collaboration with CMH leadership, multiple components of the CLF intervention were refined to enhance its relevance, feasibility, and fit for consumers with SSDs. Modifications were made to the CLF provider training materials, treatment protocol, and consumer take-home materials. Key modifications included: (1) developing a psychosis-specific suicide risk factor checklist to support individualized dialogue about symptoms; (2) creating a visual aid to guide discussion of the non-linear nature of psychosis recovery and associated vulnerability points; (3) preparing providers to address ambivalence toward calling 988 related to stigma, prior negative treatment experiences, and concerns about autonomy; (4) incorporating structured discussion on how stigma shapes help-seeking; (5) establishing a comprehensive clinician training that includes education on psychosis, suicide risk, stigma, and autonomy; and (6) designing an informational brochure to increase consumer awareness and reinforce intervention content. Together, these changes position CLF for more responsive and tailored delivery in a future pilot trial.

Stakeholder themes and modification efforts point towards three notable study implications. First, suicide risk within SSDs remains relatively less understood than that of other clinical and non-clinical populations, and thus there is a need for greater provider preparation in practice. Participants overwhelmingly shared the complexities of psychosis symptoms driving suicide risk, including points of discussion exposing a lack of clarity surrounding a rationale for why and how symptoms may relate to more or less suicide thoughts and behavior. For consumers, it was expressed that people have very different experiences with how psychosis symptoms connect to suicide thinking. In alignment with prior literature (Bornheimer et al., 2024; Chalker et al., 2024), staff participants explained that much of the formal education and training of professional degree programs (e.g., psychology, social work) and in workplace environments (e.g., CMH) are often minimally focused on suicide prevention, and broadly on general populations or in relation to depression without specificity for symptom areas like psychosis. As a result, it is essential that greater efforts are made to design suicide prevention-focused training materials to include specific attention to the complexities how psychosis symptoms relate to suicide risk.

Second, given the various barriers for 988 Lifeline use are unique to SSD populations, it is essential to familiarize consumers with the 988 resource and promoting recognition of suicide warning signs proactively and *prior* to the onset of an acute episode of psychosis and/or suicide thoughts. This largely speaks to the importance of preparing CMH consumers to engage with the 988 resource through an intervention like CLF before a crisis is on the rise, as opposed to posting 988 flyers in communities with no additional introduction, context, or discussion. Participants expressed that some consumers may not understand their psychosis symptoms or what warning signs for suicide are, therefore, it can be a significant clinical challenge to reality-test when psychosis acuity increases. For example, an individual in an acute psychosis episode may experience intensifying hallucinations or delusions that may contribute to greater suspiciousness, mistrust, and social withdrawal (Haut & MacDonald, 2010; Wisman-van der Teen et al., 2022). As a result, engaging CMH consumers in an intervention that is designed to build trust and self-efficacy in engaging in crisis resources may lead to help-seeking earlier in the course of an acute episode or crisis, before psychosis symptoms may become a growing barrier for help-seeking.

Third, it is critical to consider the timing and placement of both CLF provider training and delivery within routine CMH workflows to support sustainable implementation. Stakeholders emphasized that organizational prioritization, such as leadership commitment, protected time for training, and supervisory support, is essential to support delivery. In strong alignment with the well-established implementation science literature, leadership buy-in, ongoing training, and workflow integration are key determinants of intervention adoption and fidelity in CMH settings (Aarons et al., 2014; Williams & Beidas, 2018). Participants further described that without structured systems to identify consumers to receive CLF, and particularly those with fluctuating suicide risk or psychosis symptoms, its delivery may be inconsistent or delayed until after a crisis has already escalated. To address this challenge, staff recommended embedding CLF within existing clinical touchpoints such as intake appointments, annual biopsychosocial assessments, or medication reviews. Integrating CLF into routine care not only increases feasibility but also reflects best practices for embedding suicide prevention efforts into standard mental health service structures (Rudd & Bryan, 2022; Nuij et al., 2021). By positioning CLF as a predictable and recurring component of CMH workflows, as opposed to an add-on service, CMH and other clinical settings have potential enhance provider preparedness, ensure timely delivery, and increase the likelihood of proactive CLF delivery before psychosis acuity or suicide crisis intensity becomes a barrier to help-seeking and crisis service engagement.

## Limitations

This study should be understood in the context of the following important limitations. First, findings are based on participants from a midwestern region of the United States who were primarily female, White/Caucasian, and Non-Hispanic/Latinx. As a result, it is possible there are different perceptions about the intervention and implementation barriers and facilitators in other CMH settings and among a more diverse sample of participants. Second and given the relatively small sample (*n* = 10), it was not possible to explore themes that may have emerged differently across consumers, providers, peers, and leadership participants. It was our goal to gain insights from a group of stakeholders (i.e., consumers, providers, peers, leadership) and analyze all themes that emerged across roles for a full picture of collective perspective. Third, participants learned of the intervention through a presentation, not an in-vivo experience of the intervention itself. Therefore, different modifications may be needed after a pilot trial of CLF among individuals with psychosis yields feedback on the intervention delivery and consumer experience. Lastly, all consumer participants were actively engaged in CMH services and therefore, could have been more open to the intervention than consumers more intermittently engaged or not engaged in mental healthcare services at all. Future research should focus on populations with SSDs and suicide thoughts who are not currently engaged in treatment to understand if there are unique barriers to help-seeking in those populations.

## Conclusion

Study findings emphasize the need for suicide prevention efforts in CMH that are specifically focused on crisis resource utilization like CLF, recognizing the intervention’s potential to prepare consumers for suicide crises, motivate future 988 engagement, and prevent premature suicide death. Qualitative themes of implementation barriers and facilitators informed CLF modifications, and study implications emphasize the importance of provider training, having a proactive approach to addressing crisis resource help-seeking barriers, and embedding CLF within routine CMH workflows for sustainability. CLF holds promise to deliver psychoeducation, offer engagement, and bolster motivation and readiness for future 988 use among CMH consumers with SSDs. A future pilot study is in preparation to preliminarily examine CLF for SSD consumers and future investigations will examine the effectiveness and implementation of CLF across various SSD populations and CMH geographical locations.

## Data Availability

Data are available upon request from the lead author.
